# The gradient of the reinforcement landscape influences sensorimotor learning

**DOI:** 10.1371/journal.pcbi.1006839

**Published:** 2019-03-04

**Authors:** Joshua G. A. Cashaback, Christopher K. Lao, Dimitrios J. Palidis, Susan K. Coltman, Heather R. McGregor, Paul L. Gribble

**Affiliations:** 1 Human Performance Laboratory, University of Calgary, Calgary, Alberta, Canada; 2 Hotchkiss Brain Institute, University of Calgary, Calgary, Alberta, Canada; 3 Department of Physiology and Pharmacology, Western University, London, Ontario, Canada; 4 Graduate Program in Neuroscience, Western University, London, Ontario, Canada; 5 Brain and Mind Institute, Western University, London, Ontario, Canada; 6 Department of Psychology, Western University, London, Ontario, Canada; 7 Haskins Laboratories, New Haven, Connecticut, United States of America; Johns Hopkins University, UNITED STATES

## Abstract

Consideration of previous successes and failures is essential to mastering a motor skill. Much of what we know about how humans and animals learn from such reinforcement feedback comes from experiments that involve sampling from a small number of discrete actions. Yet, it is less understood how we learn through reinforcement feedback when sampling from a continuous set of possible actions. Navigating a continuous set of possible actions likely requires using gradient information to maximize success. Here we addressed how humans adapt the aim of their hand when experiencing reinforcement feedback that was associated with a continuous set of possible actions. Specifically, we manipulated the change in the probability of reward given a change in motor action—the reinforcement gradient—to study its influence on learning. We found that participants learned faster when exposed to a steep gradient compared to a shallow gradient. Further, when initially positioned between a steep and a shallow gradient that rose in opposite directions, participants were more likely to ascend the steep gradient. We introduce a model that captures our results and several features of motor learning. Taken together, our work suggests that the sensorimotor system relies on temporally recent and spatially local gradient information to drive learning.

## Author summary

In recent years it has been shown that reinforcement feedback may also subserve our ability to acquire new motor skills. Here we address how the reinforcement gradient influences motor learning. We found that a steeper gradient increased both the rate and likelihood of learning. Moreover, while many mainstream theories posit that we build a full representation of the reinforcement landscape, both our data and model suggest that the sensorimotor system relies primarily on temporally recent and spatially local gradient information to drive learning. Our work provides new insights into how we sample from a continuous action-reward landscape to maximize success.

## Introduction

Whether a previous action is successful or unsuccessful is an important contributor to sensorimotor learning. Indeed, binary reinforcement feedback (e.g., reward) is sufficient to cause adaptation of hand aim during a reaching task, independent from error feedback [1, 2, 3, 4, 5, 6, 7]. It has been proposed that updating aim of the hand based on reinforcement feedback is model-free and occurs by sampling a continuous set of possible motor actions until one or more actions are found that improve task success [8, 9]. Sampling motor actions presumably allows the sensorimotor system to use information from the reinforcement landscape to drive adaptation.

Here we broadly define the reinforcement landscape as the mapping between all possible motor actions and the expected reward of those actions. In this context, the sensorimotor system can maximize expected reward by ascending the reinforcement landscape [10]. However, for a meaningful change in behaviour to occur there has to be an underlying process that either evaluates or accounts for whether one action is better than another. More specifically for learning to occur the sensorimotor system must account for the gradient of the reinforcement landscape, which defines the rate of change in the expected reward with respect to a change in motor action. Intuitively, the evaluation of different actions may be easier with a steeper gradient, as there would be a more salient change in the expected reward for a change in action.

The form of the reinforcement feedback influences the shape of the reinforcement landscape. Reinforcement feedback can be binary or graded, and can be provided deterministically [1, 11] or probabilistically [2, 5]. Binary reinforcement feedback signifies only whether the action was successful or unsuccessful [1, 2, 5]. Graded feedback varies the magnitude of positive feedback (reward) or negative feedback (punishment) as a function of motor action [11, 12]. Thus, the reinforcement landscape gradient can be influenced by the magnitude and or the probability of feedback. Another consideration when using graded reinforcement feedback is that humans form a nonlinear relationship between different reward (or punishment) magnitudes and their perceived value [13]. This nonlinear relationship could potentially influence how the sensorimotor system evaluates perceived changes in expected reward.

Movement variability is also thought to influence the gradient of the reinforcement landscape by creating uncertainty between intended actions and actual actions. That is, the expected reward can change depending on whether it is a function of the intended action or the actual action [10]. Further, greater movement variability has been linked to faster learning in reinforcement-based tasks as it promotes exploration of the reinforcement landscape [14, 15].

Here we designed two experiments to examine how humans adapt the aim of their hand when receiving binary reinforcement feedback. Specifically, we tested the hypothesis that the gradient of the reinforcement landscape influences sensorimotor adaptation. We manipulated the reinforcement landscape gradient by altering the expected reward (the probability of receiving reward) given the angular distance between the hand location and target. To maximize reward, participants had to update the aim of their unseen hand to a location that was not aligned with the visually displayed target. Importantly, we normalized the reinforcement landscapes to baseline movement variability on an individual basis. This normalization allowed us to assess the influence of the reinforcement landscape gradient on learning while accounting for individual differences in movement variability. We used binary reinforcement feedback to eliminate the potentially confounding nonlinear relationship between different magnitudes of reward and their perceived value.

We tested the prediction that a steep reinforcement landscape would lead to faster learning than a shallow landscape (**Experiment 1**). Building on these results, in **Experiment 2** we used a complex reinforcement landscape where each participant’s initial action was positioned in the ‘valley’ between two slopes that had different gradients (steep and shallow) and rose in the opposite direction (clockwise or counterclockwise). We predicted that participants would ascend the steeper portion of the complex reinforcement landscape. Finally, we introduce a model that relies on binary reinforcement feedback to update the aim of the hand during a reaching task.

## Results

### Experimental design

**Fig 1:**
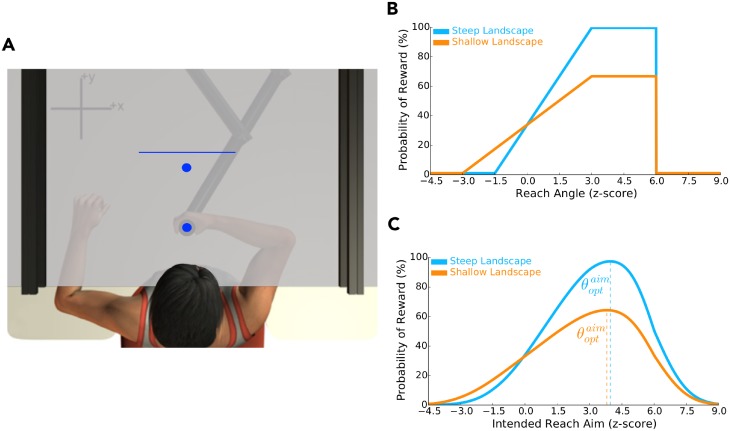
Apparatus and Experiment 1 design. **A)** Participants held the handle of a robotic arm. A semi-silvered mirror reflected images (target and home position) from an LCD screen onto a horizontal plane aligned with the shoulder, and occluded vision of the hand and arm. Participants made forward reaches from a home position, attempted to pass through a virtual target, and stopped once they passed over a horizontal line that disappeared when crossed. We informed participants that they would receive positive reinforcement for each target hit (target expanded, pleasant noise, and monetary reward). Unbeknownst to them, we manipulated **B)** the reinforcement landscape (steep or shallow), which dictated the probability of receiving reward (*y*-axis) as a function of their reach angle (*x*-axis). Reach angle was calculated relative to the line that intersected the target and home position, where the latter was the centre of rotation. To control for individual differences in movement variability, these reinforcement landscapes were scaled according to baseline reach behaviour. Accordingly, a z-score of 0.0 corresponds to their average baseline reach angle. A z-score of 1.0 corresponds to a reach angle that was 1.0 standard deviation, relative to baseline movement variability, away from the average baseline reach angle. We expected participants to adjust their aim such that they moved from their average baseline reach angle (0.0 z-score) to one that produced greater reward (z-score between 3-6). Critically, we predicted that participants experiencing the steep (blue) reinforcement landscape would learn to adjust their reach aim at faster rate than those experiencing the shallow (orange) reinforcement landscape (**Experiment 1**). **C)** Using Eqs 7–9, we accounted for movement variability to calculate the probability of reward (*y*-axis) given intended reach aim (*x*-axis). The blue and orange vertical dashed lines correspond to the optimal intended reach aim ($\theta_{opt}^{aim}$, Eq 10) that maximized the probability of reward for the steep and shallow reinforcement landscapes, respectively.

In **Experiments 1** and **2**, 120 participants performed 450 forward reaching movements (Fig 1A). For each trial they began at a starting position and attempted to pass their hand (unseen) through a virtually displayed target. We recorded reach angle, which was calculated relative to the line that intersected the visually displayed target and starting position, the moment their hand was 20 cm away from the starting position.

Participants began by completing 50 baseline trials, where no feedback was received on whether reaches were successful or unsuccessful. During the next 350 experimental trials participants received binary reinforcement feedback according to their randomly assigned reinforcement landscape (see **Experiment 1** and **Experiment 2**). Like baseline, the final 50 washout trials were also performed without feedback.

We instructed participants to “hit the target”. We informed participants that no feedback would be received if they missed the target, and for each target hit 1) the target would expand, 2) they would hear a pleasant noise, and 3) they would receive monetary reward, such that they could earn up to $5.00 CAD.

To test the idea that the gradient of the reinforcement landscape influences sensorimotor learning, we manipulated the probability of receiving positive reinforcement feedback (i.e., reward) as a function of reach angle. In **Experiment 1** we tested the idea that the gradient of the reinforcement landscape would influence the rate of learning. In **Experiment 2** we tested the notion that the sensorimotor system would use gradient information from a complex reinforcement landscape to find the best of multiple solutions that improved performance.

### Experiment 1

We tested the idea that the gradient of the reinforcement landscape influences the rate of learning. We predicted that a steeper reinforcement landscape would lead to a faster learning rate.

Participants either experienced a steep reinforcement landscape (*n* = 40) or a shallow reinforcement landscape (*n* = 40). To control for direction, the probability of positive reinforcement (reward) rose either in the clockwise (Fig 1B; Eq 2) or counterclockwise direction (Eq 3). We created these landscapes by manipulating the probability of reward as a function of reach angle. The width of each reinforcement landscape, that is the probability of reward given reach angle, was normalized to baseline movement variability on an individual basis. This normalization ensured that participants in an experimental group (steep or shallow) experienced the same gradient for a particular landscape, irrespective of movement variability. This also allowed us to calculate the change in reward probability for a change in intended aim (Fig 1C, Eqs 7–9) across participants, as well as the optimal intended reach aim ($\theta_{opt}^{aim}$) that maximized success (Eq 10).

Reach angles were normalized by baseline movement variability on an individual basis and expressed as a z-score. Further, to allow for visual and statistical comparison irrespective of the direction that the reinforcement landscape rose (clockwise or counterclockwise), we multiplied the normalized reach angles by −1.0 for all participants that experienced a reinforcement landscape that increased in the counterclockwise direction [5, 16].

Similar to others [17, 18], we found two subpopulations of participants in **Experiment 1**: learner and non-learners. When examining the histogram of final reach position (average normalized reach angle of the last 100 experimental trials), we found a bimodal distribution (S1 Data, S1 Fig). Based on this analysis, we found that a cutoff z-score of 1.0 did well to partition the bimodal distribution and separate the learners from the non-learners.

**Fig 2:**
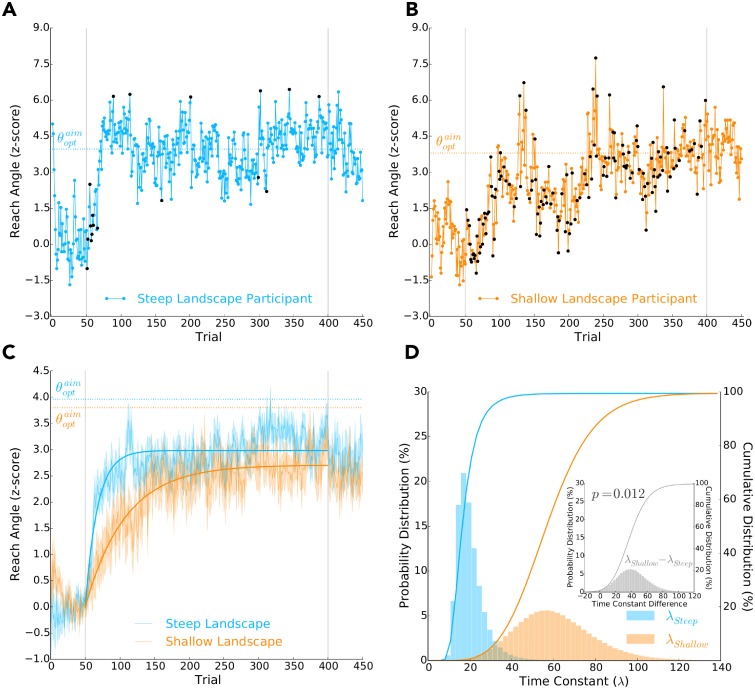
Behavioral data of Experiment 1. Reach angle (*y*-axis) over trials (*x*-axis): **A)** of a participant that experienced the steep reinforcement landscape, and **B)** of a participant that experienced the shallow reinforcement landscape, and **C)** averaged across participants that experienced either the steep (blue) or shallow (orange) reinforcement landscape, where shaded regions represent ± 1.0 SE. The grey vertical lines separate baseline trials (1-50), experimental trials (51-400) and washout trials (401-450). The dashed horizontal lines indicate the optimal intended reach aim ($\theta_{opt}^{aim}$) to maximize reward. In **A)** and **B)**, during the experimental trials, the blue and orange circles respectively indicate that the participant received reward on a given trial, while the black circles indicate no reward. In **C)**, the thick blue and orange curves represents the best-fit exponential functions to the reach angles of participants that experienced the steep or shallow reinforcement landscapes, respectively. The time constant (λ) of these exponential functions characterize the rate of learning and were found via a bootstrapping procedure. **D)** Posterior probability distributions of the exponential function time constants given the experimental reach angles (left *y*-axis) of participants that experienced the steep (λ_*steep*_, blue) or shallow (λ_*shallow*_, orange) reinforcement landscapes. The thick lines are the corresponding cumulative distributions (right *y*-axis). The inset represents the posterior probability distribution of the time constant differences between the shallow and steep participants (i.e., λ_*shallow*_ − λ_*steep*_). As observed in **C)** and **D)**, we found that participants who experienced a steep reinforcement landscape had a significantly faster rate of learning (i.e., λ_*steep*_ < λ_*shallow*_) than those that experienced a shallow landscape (*p* = 0.012).

Fig 2A and 2B shows individual data from two participants. The participant experiencing a steep reinforcement landscape quickly changed their behaviour towards a reach angle that maximized reward (z-score between 3 and 6). The participant experiencing a shallow reinforcement landscape took comparatively longer to change their reaching behaviour. The difference in learning rates between these two participants is most evident during the first 50 experimental trials.

Fig 2C shows the average reach angle over trials for participants (learners) that experienced either a steep or shallow reinforcement landscape. To compare the rate of learning between these two groups of participants, we fit an exponential function (Eq 6) over the experimental trials via bootstrapping (see Methods for details). We were interested in the time constant of the exponential function, λ, which defines the rate of learning. The exponential bootstrap fit analysis was performed separately first with the data from the learners alone, and then again with all participants (learners and non-learners together). As hypothesized, we found that the participants experiencing the steep landscape had faster learning (i.e., a lower exponential function time constant, λ) than those experiencing a shallow reinforcement landscape (*p* = 0.012 learners only, *p* = 0.021 for combined learners and non-learners, one-tailed). Fig 2D shows the posterior probability distribution and cumulative distribution of the time constant λ given the reach angles of participants experiencing either a steep or shallow reinforcement landscape. The inset of Fig 2D shows the posterior probability distribution of the time constant difference between the two experimental groups, from which we calculated the *p*-values reported directly above. The direction of the reinforcement landscape, clockwise or counterclockwise, did not influence the rate of learning (*p* = 0.540, two-tailed).

We also found that participants who experienced a steep landscape were more likely to be classified as learners than those who experienced a shallow reinforcement landscape (*p* = 0.036, two-tailed; Table 1).

**Table 1:** Frequency of learners and non-learners. Frequency of learners and non-learners partitioned based on whether the participants experienced a steep or shallow reinforcement landscape.

Group	Learners	Non-Learners
Steep Reinforcement Landscape	37	3
Shallow Reinforcement Landscape	29	11

### Experiment 2

In this experiment we tested the notion that the sensorimotor system uses gradient information from a complex reinforcement landscape to find the solution that maximizes reward. The probability of reward was at a minimum for reaches toward mean baseline behaviour but increased at different gradients (steep or shallow) for reaches in either direction (clockwise or counterclockwise) away from the target. We predicted that a significantly greater number of participants would adapt their reach aim in the direction of the steeper gradient.

**Fig 3:**
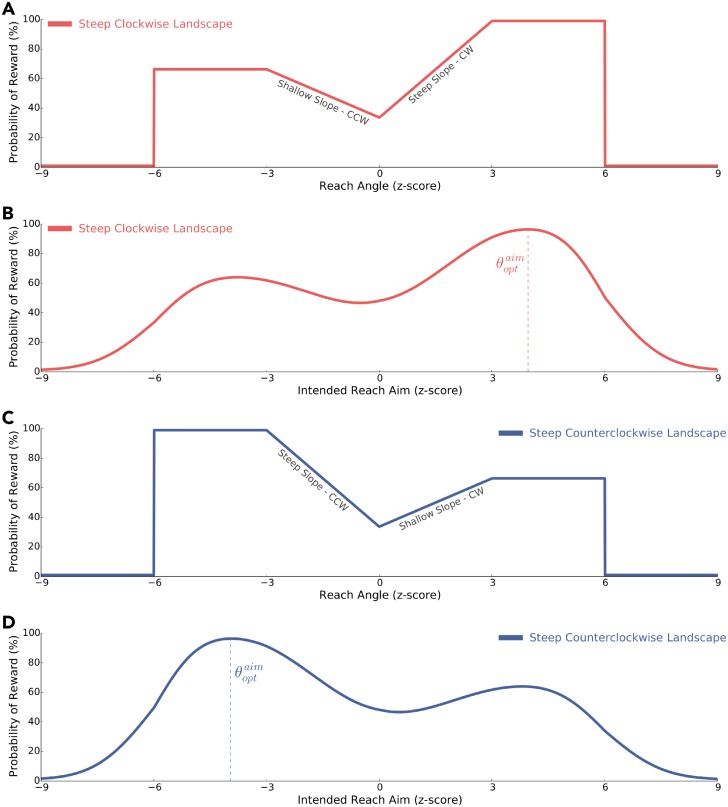
Experimental 2 design. Participants experienced a complex reinforcement landscape where they were initially positioned in the ‘valley’ between a steep and shallow slope. Participants experienced a reinforcement landscape where the steep slope rose in the **A)** clockwise direction (CW) or **C)** counterclockwise (CCW) direction. These reinforcement landscapes define the probability of reward (*y*-axis) as a function of reach angle (*x*-axis). Reach angle is normalized to baseline reach behaviour and is expressed as a z-score. **B)** and **D)** define the probability of reward (*y*-axis) given intended aim (*x*-axis) for the steep clockwise and steep counterclockwise reinforcement landscapes, respectively. In both these figures, $\theta_{opt}^{aim}$ and the corresponding dashed vertical line correspond to the optimal intended reach aim that maximizes reward. We predicted that participants experiencing the steep clockwise reinforcement landscape to adjust their aim in the clockwise direction. Similarly, we expected that those experiencing the steep counterclockwise reinforcement landscape to adjust their aim in the counterclockwise direction.

Two different reinforcement landscapes were used in this experiment: one landscape had a steep slope that rose in the clockwise direction and a shallow slope that rose in the counterclockwise direction (steep clockwise; *n* = 20; Fig 3A; Eq 4), and the other landscape had a steep slope that rose in the counterclockwise direction and a shallow slope that rose in the counterclockwise direction (steep counterclockwise; *n* = 20; Fig 3C; Eq 5). As in **Experiment 1**, for both reinforcement landscapes we calculated the probability of reward given intended aim (Fig 3B and 3D; Eqs 7–9), as well as the optimal intended reach aim ($\theta_{opt}^{aim}$) to maximize reward (Eq 10).

Here we were interested in the frequency of participants that changed their reach behaviour in the clockwise or counterclockwise direction, depending on whether they experienced the steep clockwise or steep counterclockwise reinforcement landscape. We used the average of the last 100 experimental trials to classify the direction of their final reach behaviour. Final reach direction was classified to be counterclockwise (z-score ≤ −1.0), center (−1.0 < z-score < +1.0) or clockwise (z-score ≥ +1.0). This classification was done separately for those experiencing a steep clockwise or steep counterclockwise reinforcement landscape.

**Fig 4:**
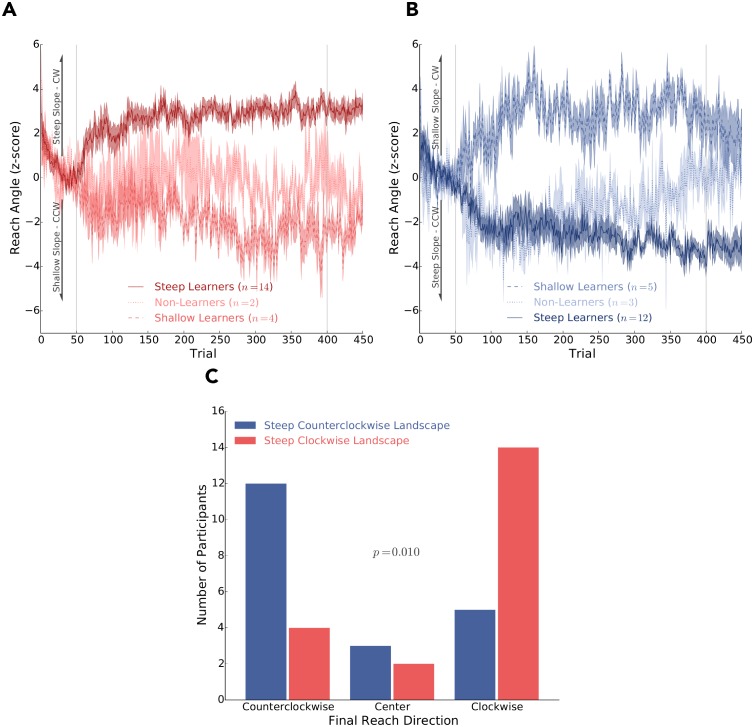
Behavioral data of Experiment 2. Average reach angles (*y*-axis) over trials (*x*-axis) of participants that experienced **A)** a steep clockwise or **B)** a steep counterclockwise reinforcement landscape. In both these subplots we partition the behaviour of participants that were classified as steep learners (solid lines), shallow learners (dashed lines), or non-learners (dotted lines). In **A)** positive reach angles correspond with the steep slope and the clockwise (CW) direction. In **B)** negative reach angles correspond with the steep slope and the counterclockwise (CCW) direction. The grey vertical lines separate baseline, experimental and washout trials. Shaded regions represent ± 1.0 SE. **C)** Frequency of participants whose final reach direction (average of the last 100 trials) was in the clockwise (z-score ≤ −1.0), central (−1.0 < z-score < +1.0), and (z-score ≥ +1.0) counterclockwise directions. As expected, we found significant differences in the frequency of final reach directions between participants that experienced the steep clockwise or steep counterclockwise reinforcement landscapes (*p* = 0.010). As predicted, we found the majority of participants used information from the complex reinforcement landscape to ascend up the steeper slope.

Fig 4A and 4B show the average reach angle of steep learners, shallow learners and non-learners for participants experiencing the steep clockwise or steep counterclockwise reinforcement landscapes, respectively. The steep and shallower learners in Fig 4A respectively look qualitatively similar to the steep and shallow learners in Fig 4B when reflecting either of these figures about its *x*-axis. The behaviour of the non-learners was less consistent based on whether they experienced the clockwise or counterclockwise landscapes, but there was a limited frequency of non-learners (*n* = 2 and *n* = 3, respectively).

As an additional classification, participants that had a final reach position corresponding to the direction of the steep slope, shallow slope or a central location, were deemed steep learners, shallow learners and non-learners, respectively. This was done separately for participants that experienced either the steep clockwise or steep counterclockwise reinforcement landscape.

For this experiment we predicted that participants would ascend the steeper gradient of their assigned reinforcement landscape. Specifically, we expected more participants who experienced the steep clockwise reinforcement landscape to have their final average reach angle to be classified as clockwise. Similarly, we expected participants who experienced the steep counterclockwise reinforcement landscape to have their final average reach angle to be classified as counterclockwise. Using z-score cutoffs of −1.0 and +1.0, we found that there were significant differences in the final average reach classification between participants who experienced a steep clockwise or steep counterclockwise reinforcement landscape (*p* = 0.010, two-tailed, Fig 4C). These results were robust to whether we used z-score cutoffs of ±0.5 (*p* = 0.016, two-tailed) or ±1.5 (*p* = 0.020, two-tailed) to classify final reach direction. Further, we found that the direction (clockwise or counterclockwise) did not influence behaviour in terms of whether a participant was classified as a steep learner, shallow learner or non-learner (*p* = 0.810, two-tailed). Thus, the direction of the reinforcement landscape had an effect on their final reach direction, but it did not impact the frequency of steep learners, shallow learners, and non-learners.

### Learning model and best-fit parameters

Here we introduce a learning model that predicts reach angle (*θ*_*n*_) on a trial-by-trial basis (Eq 1). This model takes the form

$$\begin{array}{lll} \theta_{n} & =N\left({\bar{\theta }}_{n}^{aim},\sigma_{n}^{2}\right) & \left(1a\right),\\ {\bar{\theta }}_{n+1}^{aim},\sigma_{n+1}^{2} & =\{\begin{array}{ll} {\bar{\theta }}_{n}^{aim}+\alpha \left(\theta_{n}-{\bar{\theta }}_{n}^{aim}\right),\sigma_{m}^{2} & r=1\\ {\bar{\theta }}_{n}^{aim},\sigma_{m}^{2}+\sigma_{e}^{2} & r=0 \end{array} & \begin{array}{l} \left(1b\right),\\ \left(1c\right), \end{array} \end{array}$$

where *n* and *n* + 1 represent the current and next trial, respectively.

The model considers whether the current reach angle was successful (*r* = 1) or unsuccessful (*r* = 0). The model explores small regions of the workspace in a natural way via movement variability. Here, the variance of movement variability on the current trial ($\sigma_{n}^{2}$) is a function of motor (execution) variance ($\sigma_{m}^{2}$) after a successful reach, and the addition of both motor variance and exploratory variance ($\sigma_{e}^{2}$) after an unsuccessful reach [2]. It was assumed that the variance of movement variability follows a Normal distribution $N({\bar{\theta }}_{n}^{aim},\sigma_{n}^{2})$ [19, 20, 21], where ${\bar{\theta }}_{n}^{aim}$ represents the intended reach aim on the *n*^*th*^ trial.

Inspired by Haith and Krakauer (2014) [22], the only action cached in memory is related to the location of the last successful reach. That is, an update in the intended reach aim (${\bar{\theta }}_{n}^{aim}$) occurs only after a successful reach. Specifically, this update is some proportion (*α*) of the difference between the current intended aim (${\bar{\theta }}_{n}^{aim}$) and the location of the last successful reach (*θ*_*n*_). After an unsuccessful reach, the intended aim remains the same (i.e., ${\bar{\theta }}_{n}^{aim}$ is still stored based on the last successful reach) but the subsequent movement has greater variance (*σ*_*m*_ + *σ*_*e*_). This results in a similar formulation to the equation just recently published by Therrien and colleagues (2018) [23]. There are some slight differences between the present model and the Therrien et al. (2015, 2018) model in terms of how they update the intended aim following a successful reach [23, 24] (**see** Discussion). Nevertheless, in the following we show the utility of this class of model in terms of replicating several features of sensorimotor adaptation. As previously suggested by van Beers (2009) [25] and Zhang et al. (2015) [26], our model assumes that the nervous system has some knowledge of movement variability when updating intended reach aim. This allows for an estimated difference between intended aim and actual reach angle, despite the participants have no vision of their hand during trials.

Our model has three free parameters: *α* = 0.40 (*unitless*), *σ*_*m*_ = 0.81 (z-score), and *σ*_*e*_ = 0.90 (z-score). The initial guesses of *σ*_*m*_ and *σ*_*e*_ for the fitting procedure were made with a trial-by-trial difference analysis (S2 Data, S2 Fig) that we modified from Pekny et al. (2015). It is expected that *σ*_*m*_ is slightly lower than a z-score of 1, or baseline movement variability, since here we were interested in the movement variability on a single-trial and not the additive variance that results from repeatedly subtracting two successive trials (see S2 Data, S2 Fig for further details). We found the best-fit parameters using a bootstrap optimization fitting procedure using only the data from **Experiment 1** (S3 Data).

### Simulating Experiment 1

With our learning model, we simulated 40 individuals experiencing the steep reinforcement landscape of **Experiment 1**, and then simulated another 40 individuals experiencing the shallow landscape.

**Fig 5:**
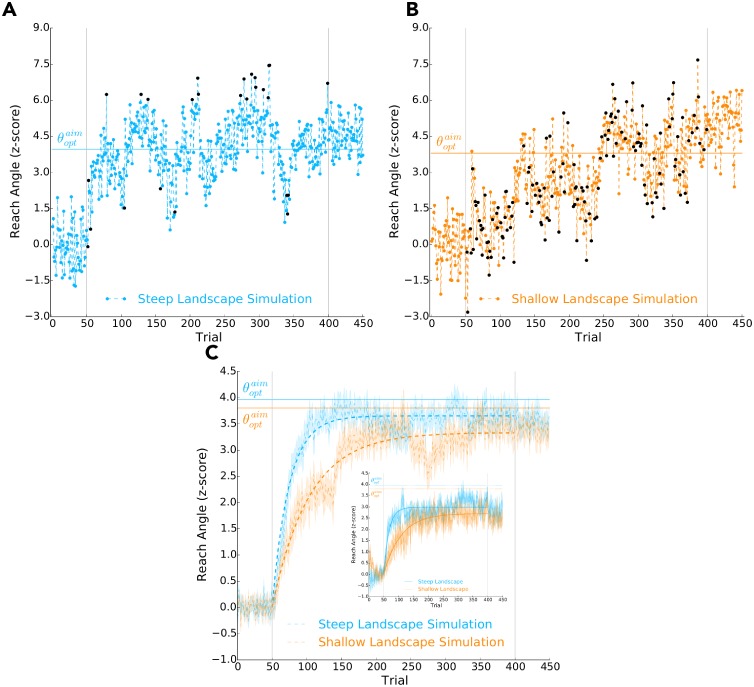
Simulations of Experiment 1. Reach angle (*y*-axis) over trials (*x*-axis) when using our learning model to simulate an ‘individual’ experiencing **A)** the steep reinforcement landscape and **B)** the shallow reinforcement landscape. In **A)** and **B)**, during the experimental trials, the blue and orange circles respectively indicate that the model received reward on a given trial, while the black circles indicate no reward. At the individual level, the learning model does well to capture individual movement variability and a faster rate of learning when experiencing the steep reinforcement landscape (compare to Fig 3A and 3B). For **A)**, **B)**, and **C)** the grey vertical lines separate baseline trials (1-50), experimental trials (51-400) and washout trials (401-450). The dashed horizontal lines indicate the optimal intended reach aim ($\theta_{opt}^{aim}$) to maximize reward. **C)** Average reach angle (*y*-axis) over trials (*x*-axis) when using the learning model to simulate 40 ‘individuals’ for both the steep (blue) and shallow (orange) reinforcement landscape. Shaded regions represent ± 1.0 SE. The thick blue and orange curves represents the best-fit exponential functions to the average reach angles of simulated ‘individuals’ that experienced the steep or shallow reinforcement landscapes, respectively. For comparison, the inset displays the behavioural data of **Experiment 1** (also shown in Fig 2C). At the group level, the learning model does well to capture between-subject variability, reproduces a faster rate of learning for the steep landscape, and gives rise to exponential learning curves.

We found that simulated individuals displayed similar trial-by-trial variance and rates of learning compared to the behavioural data (compare Fig 5A and 5B to Fig 3A and 3B). We averaged across the 40 simulated individuals in each condition (steep or shallow reinforcement landscape). The model did well to capture between-subject variance. Similar to the behavioural data, we also found the emergence of exponential learning curves (Fig 5C).

We then simulated 100, 000 individuals experiencing the steep landscape and 100, 000 individuals experiencing the shallow landscapes. Simulating a large number of individuals allowed us to numerically converge on the theoretical exponential learning curves produced by the model. We then averaged across simulated individuals in each group and fit an exponential function. The best-fit time constant, λ, of the exponential function for the steep and shallow reinforcement landscapes were 28.0 and 49.6, respectively. Both values fall within the 95^*th*^ percentile confidence intervals of the corresponding behavioural data. (steep [10.7, 36.2], shallow [27.4, 102.1]; Fig 2D).

In S2 Data, S2 Fig we present a trial-by-trial analysis, as a function of reinforcement history, of both the model simulations and behavioural data. We show in S4 Data with model simulations that changing the initial reward probability of the shallow landscape has a marginal influence on learning rates.

### Simulating Experiment 2 with the best-fit parameters found in Experiment 1

Here we simulated **Experiment 2** using our learning model (*n* = 100, 000 simulated individuals) by using the best-fit parameters obtained from the behavioural data in **Experiment 1**. To compare the model to the behavioural results, we combined the data from all participants in **Experiment 2**. This was accomplished by multiplying the normalized reach angles by −1.0 for participants that experienced the steep counterclockwise reinforcement landscape.

**Fig 6:**
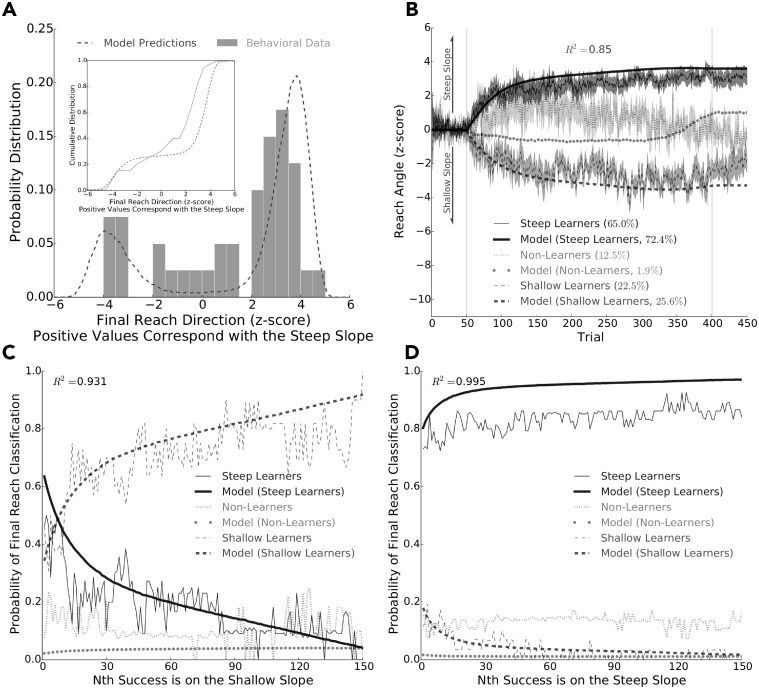
Simulations of Experiment 2 (using the best-fit parameters found in Experiment 1). **A)**, **B)**, **C)**, **D)** We simulated 100,000 ‘individuals’ experiencing the steep clockwise reinforcement landscape with our learning model. **A)** Frequency (*y*-axis) of final reach direction (*x*-axis), the average of the last 100 reaches, of the behavioural data (bars) and model (dashed line). The inset shows the corresponding cumulative distribution. As shown, we found good agreement between the model and behaviour. **B)** Reach angle (*y*-axis) over trials (*x*-axis) for the behaviour data and model outputs. Here we display the combined behavioural data of participants that experienced the steep clockwise and steep counterclockwise reinforcement landscape (see Results for details). We partitioned participants into steep learners (thin solid line), shallow learners (thin dashed line), and non-learners (thin dotted line) based on their final reach behaviour. Using the same classification criteria, the model also produced steep learners (thick solid line) and shallow learners (thick dashed line) at similar frequencies, and to some extent non-learner (thick dotted line). The model did well to capture the average reach angles of the steep learners (*n* = 26) and shallow learners (*n* = 9). It did not do well to capture the reach angles of non-learners, however there were only five participants in this group. Overall, the average reach angles of the model and behavioural data were highly correlated (*R*^2^ = 0.86). The grey vertical lines separate baseline, experimental and washout trials. Shaded regions represent ± 1.0 SE. **C)** and **D)** show the probability of becoming classified as a steep learner, shallow learner, or non-learner based based on whether the *N*^*th*^ successful reach was on the shallow slope or steep slope, respectively. Again, the participants (solid lines) and model (dashed lines) behaved similarly. These data highlight the importance of early exploration on whether an individual will maximize reward when experiencing a complex reinforcement landscape.

Fig 6A shows a histogram of the final reach angle of both the behavioural data and model simulations. We then used the same final reach direction classification for the model simulations that we used for the behavioural data. Based on these classifications, we found that the model produced a similar frequency of steep learners, shallow learners and, to some extent, non-learners as the behavioural data (Fig 6A and 6B). Further, we found that the model did well to explain reach angle over trials for these three different groups (*R*^2^ = 0.85; Fig 6B).

We also performed an analysis to explore the influence of reinforcement feedback during the initial periods of experimental trials. To this end, we calculated how a participant’s *N*^*th*^ success predicted their final reach classification. This was done separately for successful reaches made on the shallow (Fig 6C) and steep (Fig 6D) slopes of the complex reinforcement landscape. We found that if a participant had their 1^*st*^ success on the steep slope that they would likely be classified as a steep learner (Fig 6D). Conversely, a 1^*st*^ success on the shallow slope was not a good predictor of final reach classification (Fig 6D). However, a participant was likely to be classified as a shallow learner if their 15^*th*^ success and beyond was on the shallow slope. As shown, the model and data were highly correlated with each other (*R*^2^ = 0.933 and *R*^2^ = 0.995, respectively). This analysis shows that the participants and model simulations were both heavily influenced by early exploration and gradient information when they experienced a complex reinforcement landscape.

### Replicating previous work

**Fig 7:**
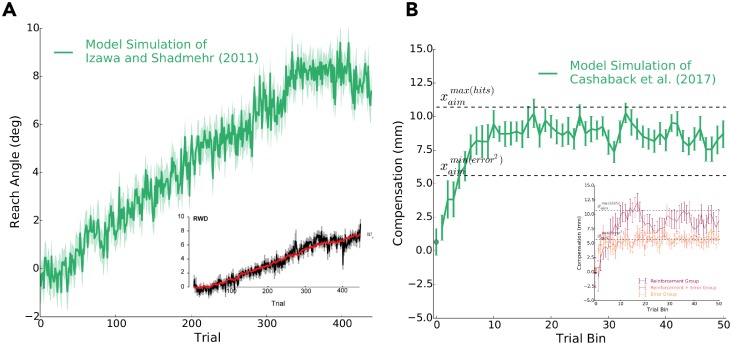
Replicating previous work using the best-fit parameters found in Experiment 1. **A)** We simulated the experiment of Izawa and Shadmehr (2011) using our learning model. Reach angle (*y*-axis) over trials (*x*-axis) as simulated by our model is shown in green (*n* = 18). The inset display the original behavioural data (black line) reported from Izawa and Shadmehr (2011; reprinted with permission). Our model captures both the linear change in reach angle and the between-subject variability. **B)** We then simulated a previous experiment of ours [5]. Hand position (i.e., compensation, *mm*) over trials (*x*-axis) as simulated by our model is shown in green (*n* = 30). The inset shows the original behavioural data, where the dark red line represents the hand position of participants when they are receiving only binary reinforcement feedback to perform the task ([5]; reprinted with permission). $x_{opt}^{max(hits)}$ represents the optimal location to aim the hand to maximize target hits (reward). Here, the model replicates the exponential learning curve, between-subject variability and suboptimal performance.

Using the same set of best-fit parameters found from the data of **Experiment 1**, we replicated the results of Izawa and Shadmehr (2011) and our previous work [5] (see Fig 7A and 7B, respectively). In the study by Izawa and Shadmehr (2011), participants were only provided binary feedback if they hit a target region that was gradually rotated from a visual displayed target. In our previous work [5], cursor position was laterally shifted according to a skewed probability distribution and participants received binary feedback on whether the laterally shifted cursor hit the visually displayed target. In both these studies, participants had no vision of their hand or arm. We had our model experience the same reported conditions from both these studies. Our model did very well to capture average reach behaviour, between-subject variance, trial-by-trial movement variability as a function of reinforcement history (see [2]; S2 Data, S2 Fig), and suboptimality.

Here, we define suboptimality as approaching but not quite reaching the optimal behaviour that maximizes reward (i.e., $x_{opt}^{max(hits)}$ in Fig 7B). Suboptimality is often a feature of ‘greedy’ algorithms that place greater emphasis on locally optimal information rather than globally optimal information [27]. Our learning model would be considered a greedy algorithm since it samples from spatially local motor actions and updates its aim based on the last recent success. A greedy algorithm can lead to suboptimal performance in non-symmetrical landscapes (e.g., [5], Fig 1B and 1C) and complex landscapes with local maximums (e.g., Fig 2). Behaviourally, this was particularly evident in **Experiment 2** where a relatively high proportion of participants (22.5%) performed suboptimally by ascending the shallow slope and having a final reach direction aligned with a local maximum.

**Fig 8:**
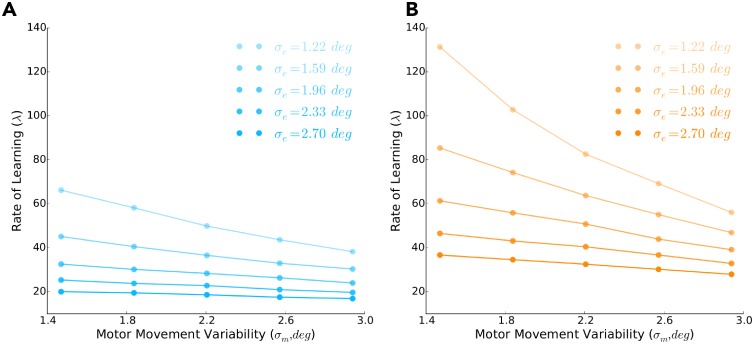
Predicting learning rates given motor and exploratory movement variability. In **A)** and **B)** we predict the rate of learning (λ; *y*-axis) after varying motor (*σ*_*m*_, *deg*; *x*-axis) and exploratory (*σ*_*e*_, *deg*; different shaded lines) contributions to movement variability, when the our learning model experiences the steep (blue) and shallow (orange) landscapes, respectively (*n* = 10, 000 per data point). In both **A)** and **B)** we find that increasing either *σ*_*m*_ or *σ*_*e*_ leads to a faster rate of learning (i.e., lower magnitudes of λ).

Further motivated by the model of Haith and Krakauer (2014) [22], we ran simulations to examine how movement variability influences the rate of learning and whether our model could capture random-walk behaviour. There is some debate to whether movement variability is beneficial [14, 22] or detrimental [15, 28, 29, 30] when learning from error feedback, which to some extent may be explained by the consistency (entropy) of the environment [31]. Recent work has suggested that movement variability is important when learning from reinforcement feedback and can influence the rate of learning [14]. Here we manipulated both motor (*σ*_*m*_) and exploratory (*σ*_*e*_) contributions to movement variability when simulating the experimental conditions of **Experiment 1**. We found that increasing the variance of movement variability, either *σ*_*m*_ or *σ*_*e*_, led to increased rates of learning for both the steep (Fig 8A) and shallow (Fig 8B) reinforcement landscapes. However, it should be noted that with different amounts of movement variability there may exist a trade-off between the rate of learning and the probability of reward.

**Fig 9:**
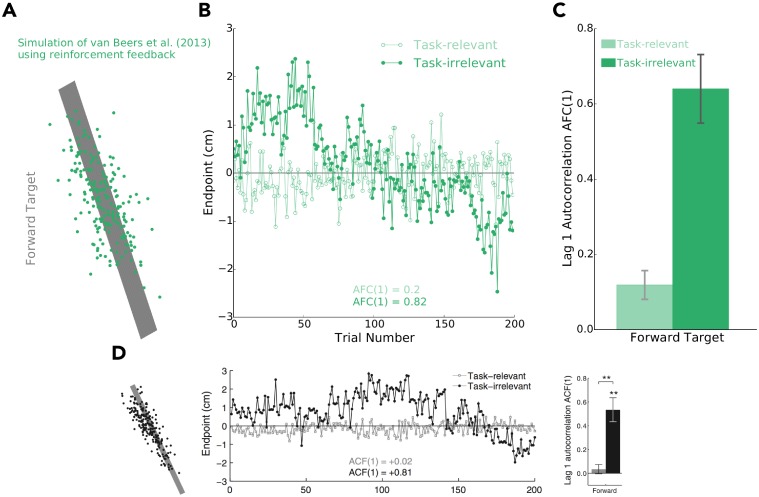
Random-walk behavior along task-irrelevant dimensions. In **A), B), C)**, we show that our learning model predicts that updating aim based on reinforcement feedback can lead to random-walk behaviour along task-irrelevant dimensions. **A)** Target with a long length (task-irrelevant dimension) and short width (task-relevant dimension). Endpoint position (green) as predicted by our model for each trial. **B)** Corresponding endpoints (*y*-axis) as partitioned into task-irrelevant (dark green) and task-relevant (light green) dimensions over trials (*x*-axis). Here *AFC*(1) represent the autocorrelation (lag 1 trial) of the task-irrelevant (dark green) and task-relevant (light green) endpoint components when simulating an individual. This autocorrelation provides insight if trials *n* and *n* + 1 are uncorrelated or correlated, where the latter is a feature of random-walk behaviour. **C)** The average AFC(1) in task-irrelevant (dark green) and task-relevant (light green) when simulating six subjects (± 1.0 SE bars). **D)** Original behavioural data from van Beers and colleagues (2013; reprinted with permission) showing task-irrelevant random-walk when participants received *error feedback*—not reinforcement feedback as simulated with our model. Although previously attributed to error-based processes, our model predicts that reinforcement-based processes may also give rise to random-walk behaviour.

In previous literature, random-walk behaviour along task-irrelevant dimensions has been attributed solely to error-based learning [32, 33, 34, 35]. In the study by van Beers and colleagues (2013), participants received error (visual) feedback when reaching to large targets (Fig 9D). They displayed random-walk behaviour (i.e., trial-by-trial correlations) along the task-irrelevant dimensions that had no bearing on task success. Here we tested whether reinforcement feedback can also lead to random-walk behaviour. To test this idea, we used our model to simulate the experiment van Beers et al. (2013). Critically however, we did not use error feedback as in the original study—instead we only provided binary reinforcement feedback to the model based on whether it had hit or missed the target. Interestingly, we found that random-walk behaviour along task-irrelevant dimensions also *emerged* from our model (Fig 9A, 9B and 9C). Thus, our simulations suggest that random-walk behaviour, at least in part, may be attributed to reinforcement-based processes.

Our model relies on updating intended reach aim by using only the recent success (temporally current information) based on sampling the reinforcement landscape via movement variability (spatially local information). Given the strong relationship between our model and the behavioural data throughout the simulations above, our results suggest that the sensorimotor system largely depends on temporally recent and spatially local information to update where to aim the hand during our reinforcement-based sensorimotor learning task.

## Discussion

We found that manipulating the gradient of the reinforcement landscape influenced sensorimotor learning. First, we found that a steep reinforcement landscape led to faster learning. Second, participants were more likely to adjust their aim in the direction of the steepest portion of a complex reinforcement landscape. Our learning model that relies on reinforcement feedback to update aim of the hand was able to replicate the results in **Experiment 1** and predict the results found in **Experiment 2**. Taken together, our data and model suggest that the sensorimotor behaviour observed in our experiments does not necessitate a full representation of the entire reinforcement landscape (storing the expected reward for all possible actions). Rather, the majority of learning behaviour can be captured using temporally recent and spatially local information about actions and rewards.

Participants learned faster when they experienced a steep reinforcement landscape, compared to those experiencing a shallow landscape. To our knowledge this is the first work showing that the gradient of the reinforcement landscape influences the rate of learning. The present study may be distinguished from previous work showing that a graded reinforcement landscape can augment error-based learning [11, 12]. Here we show that the gradient of a binary, positive reinforcement landscape influences learning in the absence of error feedback.

Using a visuomotor rotation task, Nikooyan and Ahmed (2014) used both graded reinforcement feedback and error feedback to study their effects on learning. Participants moved a cursor which was rotated from the unseen hand as it moved away from a start position towards a virtual target. Participants performed the task either with or without error (cursor) feedback. They experienced a graded reinforcement landscape, such that the magnitude of reward changed with the angular distance of the hand from the target, according to either a linear or cubic function. The maximum reward magnitude occurred when the rotated cursor hit the target. Relative to learning using only error feedback, linearly and cubically graded reinforcement landscapes combined with error feedback accelerated learning. They also found differences in the amount of adaptation between participants who experienced only graded reinforcement feedback (without any visual error feedback) based on either a linear or cubic reinforcement landscape. However, these differences reversed in direction during the course of the experiment and, in some instances, opposed theoretical predictions from a temporal-difference (TD) reinforcement algorithm [11, 36]. These inconsistent findings may have been caused by not controlling for individual differences in movement variability [14] or the nonlinear relationship between different reward magnitudes and their perceived value [13].

In our experiments, we used binary feedback that always had the same magnitude of reward. This eliminated the nonlinear relationship between different reward magnitudes and their perceived value [13]. Further, we controlled for individual differences in movement variability, which can influence exploration and the rate of learning in reinforcement-based tasks [14, 15, 37]. Thus, our work is the first to our knowledge that has isolated how the gradient of the reinforcement landscape influences the rate of sensorimotor learning.

In our second experiment, each participant’s initial action was positioned in the ‘valley’ between two slopes that had different gradients (steep or shallow) and rose in opposite directions. As predicted, we found participants were more likely to ascend the steepest portion of a complex reinforcement landscape. While the majority of participants ascended the steep slope, several participants ascended the shallow slope. The probability of whether they would be classified as a steep learner or shallow learner seemed related to initial success on either the steep or shallow portion of the landscape. In particular, participants were very likely to be classified as a steep learner if their first successful reach was on the steep slope of the complex landscape.

Our learning model did well to capture trial-by-trial behaviour, between subject variability and exponential learning curves in **Experiment 1**. Using the same set of best-fit parameters found using **Experiment 1** data, we then simulated **Experiment 2**. The model produced similar distributions of steep-learners, shallow-learners and, to some extent, non-learners. The model was also able to capture several aspects of learning reported in previous work [1, 2, 5].

As mentioned, the behavioural findings of **Experiment 2** were well predicted by our learning model. Critically, our model does not build up a full representation of the reinforcement landscape. Rather, it relies on using movement variability for spatially local exploration and temporally recent reinforcement feedback to update intended reach aim. Considering that the model does not build up a representation of the reinforcement landscape and that it was highly correlated with the behavioural results, suggests that whether participants ascended up the shallow portion or the steep portion of the complex reinforcement landscape was largely due to movement variability and the probability of reward. As an example, a participant’s initial reach angle had an equal probability of being aligned with either the steep or shallow slope due to movement variability. However, a participant’s initial reach was more likely to be rewarded on the steep slope because of its higher rate of reward. Moreover, the further a participant ascended either the steep or shallow slope it became increasingly unlikely that future successes would promote them from descending a slope. In particular, the steep slope had a stronger effect of promoting participants to ascend since its reward rate was double that of the shallow slope. This is evident in Fig 6D, where both the participants and model simulations were very likely to be classified as a steep learner when they had their 1^*st*^ success on the steep slope. Conversely, final reach classification for both the participants and model simulations only became reliable after approximately the 15^*th*^ success on the shallow slope (Fig 6C). Thus, participants and the model were more likely to be initially rewarded on the steep slope and also more likely to ascend the steep slope. Taken together, our behavioural results and model simulations support the idea that the nervous system does not build up a representation of the reinforcement landscape. Rather, the nervous system seems to rely on spatially local movement variability for exploration and temporally recent reinforcement feedback to update hand aim. Importantly, our findings also suggest that early exploration is highly influential when attempting to avoid local maximums and discover a global maximum.

Several hallmarks of motor learning simply emerged from our phenomenological learning model. Specifically, we found that the model produces exponential learning curves, between-and within-subject movement variability, suboptimal performance, increased learning rates with greater movement variability, trial-by-trial variance given a successful or unsuccessful reach (S2 Data, S2 Fig), reduced variability when hand aim approaches the optimal solution to maximize success, and random-walk behaviour in task-irrelevant dimensions. To our knowledge, random-walk behaviour has only been previously associated with error-based learning [32, 33, 34, 35]. Future work should examine whether random-walk behaviour can be replicated with experiments involving only reinforcement feedback.

The model of Haith and Krakauer (2014) [22] and the recently published model of Therrien and colleagues (2018) [23] would also be able to reproduce the rich set of behavioural phenomena mentioned in the above paragraph. These two models also rely on movement variability for exploration and caching a single aim direction that can be updated based on recent feedback. The Haith and Krakauer model stems from a Markov chain Monte Carlo (MCMC) algorithm and relies on sampling different motor actions. Actions are drawn from a probability distribution with a previously cached action acting as the distribution mean. If a recently experienced action is deemed less costly and or more rewarding than the previously cached action, this recent action becomes the newly cached action. Although this model was demonstrated with error-based tasks (i.e., visuomotor rotation and force-field adaptation), it could be extended to update hand aim using reinforcement feedback. As mentioned above, the work of Haith and Krakauer (2014) [22] and Pekny et al. (2015) [2] provided the motivation for our model. This resulted in a similar set of equations as recently proposed by Therrien and colleagues (2018) [23], albeit with some slight differences in terms of how the model updates hand aim. In their model, updating hand aim relies on the assumption that the sensorimotor system has perfect knowledge of additional exploratory movement variability following an unsuccessful reach and partial knowledge of the motor (execution) variability following a successful reach. Conversely, our model assumes that the same proportion of motor and exploratory movement variability are known by the sensorimotor system when updating hand aim. While some studies have explored the idea that the sensorimotor system has some awareness of movement variability [25, 26], to our knowledge no study has explored what proportion of movement variability is known by the sensorimotor system following a successful or unsuccessful reach. Nevertheless, our present work highlights the utility of this class of models, which rely on movement variability for exploration and caching a single action, to predict sensorimotor adaptation.

Emergent behaviour and simplicity are perhaps the most attractive features of our learning model. The model uses movement variability to sample the reinforcement landscape locally, and temporally recent information to update where to aim the hand. These features distinguish our model from several mainstream reinforcement algorithms in the motor literature that rely on building a full representation of the reinforcement landscape [1, 11, 37, 38]. The explicit goal of these algorithms is to maximize reward. For many of these reinforcement learning models, exploration and maximizing reward is accomplished by selecting actions using a soft-max function that considers the expected value of all possible actions. In general, such models rely on a large number free parameters and assumptions. Depending on the task and the discretization of considered actions and states, storing a representation of the reinforcement landscape in real-world situations could require vast amounts of memory and may be implausible. In comparison, our model (similarly, [22, 23]) has a small number of free parameters, makes few assumptions, implicitly maximizes reward, and uses minimal memory.

Our learning model does well to capture several aspects of behaviour during learning. For the model to adapt however, there has to be a non-zero gradient within the range of naturally occurring movement variability. Thus, the model is limited to small areas of the workspace. It has been shown in previous studies that participants are unaware of a change in aim when operating over small areas of the workspace [1, 39]. In our task, the average change in behaviour was ∼ 7.0 degrees, suggesting that the participants in our experiments were also likely unaware of the small shifts in reach angle [40]. Learning beyond these small areas of the workspace would likely also require active (cognitive) exploration strategies [41] and explicit awareness of the reinforcement landscape [17]. Nonetheless, our model did well to capture many features of sensorimotor adaptation over small areas of the workspace.

Behaviourally, we found that a steeper reinforcement landscape leads to faster learning. We also found that humans are more likely to ascend the steepest portion of a complex landscape. Our model was able to replicate our findings without the need to build up a representation of the reinforcement landscape. Further, several hallmarks of human learning simply emerged from this model. Taken together, our data and our model suggest that the sensorimotor system may not rely on building a representation of the reinforcement landscape. Rather, over small areas of the workspace, sensorimotor adaptation in reinforcement tasks may occur by using movement variability to locally explore the reinforcement landscape and recent successes to update where to aim the hand.

## Methods

### Participants

80 individuals participated in **Experiment 1** (20.1 *years* ± 2.8 SD) and 40 individuals participated in **Experiment 2** (20.5 *years* ± 2.8 SD). Participants reported they were healthy, right-handed and provided informed consent to procedures approved by Western University’s Ethics Board.

### Apparatus

In both experiments, participants held the handle of a robotic arm (InMotion2, Interactive Motion Technologies, Cambridge, MA; Fig 1A) and made right-handed reaching movements in a horizontal plane. An air-sled supported each participant’s right arm while providing minimal friction with the desk surface during the reaching movements. A semi-silvered mirror blocked vision of both the participant’s upper-limb and the robotic arm, and projected images from an LCD screen onto a horizontal plane passing through the participant’s shoulder. An algorithm controlled the robot’s torque motors and compensated for the dynamical properties of the robotic arm. The position of the robotic handle was recorded at 600 *Hz* and the data were stored for offline analysis.

### Protocol

#### Reaching task for Experiment 1 and 2

Participants were presented with virtual images of a start position (0.5 cm diameter, blue circle), a target (0.5 cm diameter, blue circle) located 20 cm forward of the start position, and a blue finish line located 2 cm beyond the target (Fig 1A). For each trial, participants began from a start position, passed by or through the target, and then stopped their reach when the robot handle passed over the finish line that disappeared once crossed. After 1 sec, the finish line reappeared and the robotic arm returned the participant’s hand to the starting position.

Participants performed 450 reaching movements. We instructed them to “hit the target”. Participants received no feedback during baseline reaches (trials 1 − 50). During the experimental reaches (trials 51 − 400), they received binary reinforcement feedback that was dependent on their assigned reinforcement landscape. We told them that each time they hit the target: 1) it would expand (5*x*) in diameter, 2) they would hear a pleasant noise, and 3) that they could earn monetary reward. Participants were informed that they could earn up to 5.00 CAD based on their performance. We also told participants that if they missed the target, no feedback would be presented and the robot would return them to the start position after they passed the finish line. During washout (trials 401 − 450) participants received no feedback.

### Reinforcement landscapes

During both experiments, participants were exposed to one of several different reinforcement landscapes. We manipulated the gradient of the reinforcement landscapes by controlling the probability of positive reinforcement (reward) as a function of reach angle. These landscapes were constructed such that participants had to learn to change their reach angle, relative to baseline performance, to maximize the probability of reward.

The width of the reinforcement landscape experienced by a participant was normalized to the variability of their baseline reach angles. Reach angle was measured at the position where the robot handle first became 20 cm away from the center of the starting position, and was calculated relative to the line that intersected the starting position and the displayed target. The last 25 baseline trials were used to calculate their average baseline reach angle and the standard deviation of their angular movement variability. All reach angles were converted into z-scores. Specifically, reach angles were expressed relative to the average baseline reach angle and then normalized by the participant’s average standard deviation recorded during baseline. Thus, a z-score of 0.0 corresponded with their average baseline reach angle. A z-score of 1.0 or −1.0 indicated that a reach angle was ± 1 SD away from their average baseline reach angle in the clockwise or counterclockwise direction, respectively.

Defining the reinforcement landscape in terms of a z-score served two purposes. First, we controlled for slight differences in individual aiming bias by positioning all participants on the same location of the reinforcement landscape during the start of the experimental trials. Second, we normalized the width of the reinforcement landscape for each participant based on baseline movement variability, allowing us to isolate how the reinforcement landscape gradient influenced learning.

#### Experiment 1

Here we tested the idea that the gradient (steep or shallow) of a reinforcement landscape influences the rate of learning. As a control, we also manipulated the direction that the reinforcement landscape increased along (clockwise or counterclockwise). Testing both directions assured us that changes in behaviour were not caused by systematic drift across trials. These manipulations resulted in four different reinforcement landscapes: a steep landscape increasing in the clockwise direction (Fig 1B), a shallow landscape increasing in the clockwise direction (Fig 1B), a steep landscape increasing in the counterclockwise direction, and a shallow landscape increasing in the counterclockwise direction. We predicted that participants would have faster learning in the steep condition relative to the shallow condition. Participants were pseudorandomly assigned to one of the four reinforcement landscapes (*n* = 20 participants per condition).

For the four reinforcement landscapes, average baseline behaviour (0.0 z-score) led to a 33.0% probability of receiving positive reinforcement. The probability of reward in the steep clockwise $\left(R{\left(\theta \right)}^{CW_{steep}}\right)$ and shallow clockwise $\left(R{\left(\theta \right)}^{CW_{shallow}}\right)$ reinforcement landscapes rose in the clockwise direction (*CW*) at a rate of 22.2% per z-score and 11.1% per z-score, respectively. These two reinforcement landscapes, which define the probability of success given reach angle [*p*(*r* = 1|*θ*)], can be summarized with

$$\begin{array}{cc} R{\left(\theta \right)}^{CW_{i}}=p{\left(r=1|\theta \right)}^{CW_{i}} & =\{\begin{array}{ccc} 0; & \theta <-\frac{3}{3\cdot m_{i}-1} & \left(2a\right),\\ \left(\frac{m_{i}}{3}-\frac{1}{9}\right)\cdot \theta +\frac{1}{3}; & -\frac{3}{3\cdot m_{i}-1}\leq \theta \leq 3 & \left(2b\right),\\ m_{i}; & 3<\theta \leq 6 & (2c),\\ 0; & \theta >6 & (2d). \end{array} \end{array}$$

*r* = 1 denotes a successful reach. The maximal success rate, *m*_*i*_, was between 3.0 to 6.0 z-score away from the average baseline reach angle in the clockwise direction. More specifically, *m*_*steep*_(1.0) and *m*_*shallow*_(2/3) define the maximal success rate of the steep and shallow landscapes, and are used to calculate both the landscape slopes and *x*-intercepts. Along the counterclockwise direction, the probability of success decreased linearly until 0.0%. Elsewhere, the probability of success was 0.0%. *θ* is expressed as a z-score.

The steep counterclockwise ($R{\left(\theta \right)}^{CCW_{steep}}$) and shallow counterclockwise ($R{\left(\theta \right)}^{CCW_{shallow}}$) reinforcement landscapes are mirror images, reflected about the average baseline reach angle (0.0 z-score), of their clockwise counterparts. They are summarized as

$$\begin{array}{cc} R{\left(\theta \right)}^{CCW_{i}}=p{\left(r=1|\theta \right)}^{CCW_{i}} & =\{\begin{array}{ccc} 0; & \theta <-6 & (3a),\\ m_{i}; & -6\leq \theta <-3 & (3b),\\ \left(\frac{1}{9}-\frac{m_{i}}{3}\right)\cdot \theta +\frac{1}{3}; & -3\leq \theta \leq \frac{3}{3\cdot m_{i}-1} & \left(3c\right),\\ 0; & \theta >\frac{3}{3\cdot m_{i}-1} & \left(3d\right). \end{array} \end{array}$$

#### Experiment 2

Here, we tested the idea that the sensorimotor system is able to use local gradient information to ascend the steepest slope of a complex reinforcement landscape. To investigate, participants were initially positioned between two slopes that rose at differing rates (steep and shallow) and opposite directions (Fig 3A and 3C). We tested two landscapes: 1) a steep clockwise condition (Fig 3A); where the steeper slope of the reinforcement landscape rose in the clockwise direction and the shallow slope rose in the counterclockwise direction and 2) a steep counterclockwise condition (Fig 3C); where the steeper slope of the reinforcement landscape rose in the counterclockwise direction and the shallow slope rose in the clockwise direction. We predicted that a greater proportion of participants would ascend the steeper gradient, irrespective of direction (clockwise or counterclockwise). Similar to **Experiment 1**, we used two directions (steep clockwise or steep counterclockwise) to be assured that changes in behaviour were not due to systematic drift. Participants were pseudorandomly assigned to one of these two reinforcement landscapes (*n* = 20 participants per condition).

The steep clockwise condition (*R*(*θ*)^*StCW*^) can be summarized with

$$\begin{array}{cc} R{\left(\theta \right)}^{StCW}=p{\left(r=1|\theta \right)}^{StCW} & =\{\begin{array}{ccc} 0; & \theta <-6 & (4a),\\ \frac{2}{3}; & -6\leq \theta <-3 & (4b),\\ -\frac{1}{9}\cdot \theta +\frac{1}{3}; & -3\leq \theta <0 & (4c),\\ \frac{2}{9}\cdot \theta +\frac{1}{3}; & 0\leq \theta \leq 3 & (4d),\\ 1; & 3<\theta \leq 6 & (4e),\\ 0; & \theta >6 & (4f). \end{array} \end{array}$$

The gradients of the steep and shallow slopes were identical to those described in **Experiment 1**. The maximal success rate (100.0%) in the clockwise direction occurred between 3.0 to 6.0 z-score, while the maximal success rate (66.7%) in the counterclockwise direction occurred between −3.0 to −6.0 z-score in the counterclockwise direction. Elsewhere, the probability of success was 0.0%.

The steep counterclockwise condition (*R*(*θ*)^*StCCW*^) was the mirror image of the steep clockwise condition, reflected about the average baseline reach angle (0.0 z-score). This is summarized by

$$\begin{array}{cc} R{\left(\theta \right)}^{StCCW}=p{\left(r=1|\theta \right)}^{StCCW} & =\{\begin{array}{ccc} 0; & \theta <-6 & (5a),\\ 1; & -6\leq \theta <-3 & (5b),\\ -\frac{2}{9}\cdot \theta +\frac{1}{3}; & -3\leq \theta <0 & (5c),\\ \frac{1}{9}\cdot \theta +\frac{1}{3}; & 0\leq \theta \leq 3 & (5d),\\ \frac{2}{3}; & 3<\theta \leq 6 & (5e),\\ 0; & \theta >6 & (5f). \end{array} \end{array}$$

### Data analysis

We performed data analysis using custom Python 2.7.11 scripts. For all participants in both Experiments, we recorded their endpoint reach angle for each of the 450 trials. Reach angles were normalized based on baseline reach behaviour, as described above, and expressed as a z-score.

#### Experiment 1

To perform comparisons across groups, we multiplied the normalized reach angles by −1.0 for all participants experiencing a reinforcement landscape that increased in the counterclockwise direction [5, 16].

Here we were primarily interested in the rate of learning given the gradient (steep or shallow) of the assigned reinforcement landscape. The rate of learning is captured in the λ term of the following exponential function [42]:

$$\begin{array}{c} \theta_{i}=a\left(1-e^{-i/\lambda }\right), \end{array}$$ (6)

where *θ*_*i*_ is the estimated reach angle (z-score) on the *i*^*th*^ experimental trial, *e* (2.71) is a constant, and *a* defines the asymptotic reach angle (z-score). We used least squares to fit this equation to the experimental trials (51 to 400) via bootstrapping. Specifically, we fit an exponential function for each bootstrap resample, allowing use to estimate the posterior distribution of each parameter given the data. The bootstrapping technique also allowed for statistical comparison to be made between the two groups. We expected participants experiencing a steep reinforcement landscape to learn faster (i.e., have a significantly lower λ) than those experiencing a shallow landscape.

When inspecting individual data, there seemed to be two distinct subpopulations of participants: learners and non-learners. For all participants in **Experiment 1**, we characterized their asymptotic reaching behaviour by calculating their average reach angle during the last 100 trials of the experimental trials. We found that a final reach angle of 1.0 z-score was an appropriate cutoff to separate these two subpopulations (S1 Data, S1 Fig). We then summed the number of learners and non-learners based on whether they experienced a shallow or steep reinforcement landscape (Table 1).

#### Experiment 2

In this experiment, we were interested in the final reach direction after participants had been initially positioned between a shallow slope and steep slope acting in opposite directions. We averaged the last 100 experimental trials to calculate each participant’s asymptotic behaviour. We then classified each participant’s final asymptotic reach behaviour using the same cutoff used in **Experiment 1**. Specifically, final reach behaviour was classified to be counterclockwise (z-score ≤ −1.0), center (−1.0 < z-score < +1.0) or clockwise (z-score ≥ +1.0). Separately for those experiencing a steep clockwise or steep counterclockwise reinforcement landscape, we counted the number of participants whose asymptotic reach behaviour fell into these classifications.

We predicted that participants would ascend the steeper slope of the complex landscape. Consequently, we expected significant differences in the final average reach classification between participants that experienced a steep clockwise or steep counterclockwise reinforcement landscape. As a reminder, final reach position was calculated as the average of the last 100 experimental trials. For all participants in **Experiment 2**, those who had a final reach position corresponding to the direction of the steep slope, shallow slope or a central location were termed: steep learners, shallow learners and non-learners, respectively.

We also performed an analysis to explore the influence of reinforcement feedback during the initial periods of the experimental trials. To this end, we calculated how the location (steep or shallow slope) of their *N*^*th*^ success predicted the likelihood of their final reach classification. This analysis provides insight into the influence of both early exploration and gradient information on how a complex reinforcement landscape is experienced over the course of learning.

#### Probability of reward given intended reach aim for Experiment 1 and 2

For all experimental conditions, we calculated the probability of reward given the intended reach aim (Fig 1C; Fig 3B and 3D). Critically, this analysis demonstrates that the experimentally imposed reinforcement landscapes still lead to different gradients (steep or shallow) when accounting for normalized movement variability.

The probability of reward, that is the expected utility (*E*[*U*_Θ_(⋅)]) for the set of possible actions (Θ), is estimate by solving

$$\begin{array}{c} E\left[U_{\Theta }\left({\bar{\theta }}^{aim},\sigma^{2}\right)\right]=\int p\left(\theta |{\bar{\theta }}^{aim},\sigma^{2}\right)R{\left(\theta \right)}^{j}d\theta , \end{array}$$ (7)

where [*R*(*θ*)^*j*^] is the experimentally imposed reinforcement landscape and [$p(\theta |{\bar{\theta }}^{aim},\sigma^{2})$] is the probability of some reach angle [19, 43, 44].

Reach angle (*θ*) was modelled with a Normal distribution [19, 20, 21],

$$\begin{array}{c} p\left(\theta |{\bar{\theta }}^{aim},\sigma^{2}\right)=\frac{1}{\sigma \sqrt{2\pi }}\;e^{-\frac{{\left(\theta -{\bar{\theta }}^{aim}\right)}^{2}}{2\sigma^{2}}}, \end{array}$$ (8)

where *θ*^*aim*^ represents an unbiased aim and *σ*^2^ is the overall reach angle variance. *σ*^2^ was estimated by considering both motor (execution) variance ($\sigma_{m}^{2}$) and exploration variance ($\sigma_{e}^{2}$) [2, 25]. Pekny and colleagues (2015) proposed that the magnitude of exploration variability is inversely related to the probability of reward, the latter of which we manipulated as a function of reach angle [*p*(*r* = 1|*θ*)^*j*^] according to the assigned reinforcement landscape (*j*). Thus, by considering two potential sources of movement variability and the probability of reward, *σ*^2^ was approximated by the following equation:

$$\begin{array}{c} \sigma^{2}=\sigma_{m}^{2}+\left[1-p{\left(r=1|\theta \right)}^{j}\right]\cdot \sigma_{e}^{2}. \end{array}$$ (9)

Here, motor (execution) variance is constant from trial-to-trial. The influence of exploration variance scales inversely with the probability of receiving reward. The values of *σ*_*m*_(0.81) and *σ*_*e*_(0.9) were the best-fit parameters of our learning model (Eq 1). Eqs 7–9 were solved numerically by convolving the reach angle probability distribution over each of the experimentally imposed reinforcement landscapes [44].

For each reinforcement landscape, the intended reach aim that maximizes the probability of reward ($\theta_{opt}^{aim}$) corresponds to the intended reach aim that maximizes the expected reward of Eq 7. This is summarized by

$$\begin{array}{c} \theta_{opt}^{aim}=\underset{\theta^{aim}\in \Theta }{arg max}\left\{E\left[U_{\Theta }\left({\bar{\theta }}^{aim},\sigma^{2}\right)\right]\right\}. \end{array}$$ (10)

In **Experiment 1**, the $\theta_{opt}^{aim}$ for the steep and shallow clockwise reinforcement landscapes were 3.96 z-score and 3.8 z-score, respectively. In **Experiment 2**, the $\theta_{opt}^{aim}$ for the steep clockwise and the steep counterclockwise reinforcement landscapes were 3.96 z-score and −3.96 z-score, respectively.

### Statistical analysis

Tests between means were performed using bootstrapped hypothesis tests with 1, 000, 000 resamples (Python 2.7.11) [5, 45, 46, 47]. Fisher’s exact test was used to test frequency tables (R 3.2.4). Coefficient of Determination (*R*^2^) was used to compare model simulations to behavioural data (Python 2.7.11). One-sided tests were used for planned comparisons based on theory-driven predictions. For all other comparisons we used two-tailed tests. Multiple comparisons were corrected for Type-I error using the Holm-Bonferroni procedure [48]. Statistical tests were considered significant at *p* < 0.05.

## Supporting information

S1 Data Cutoff criterion used to separate learners and non-learners. Cutoff criterion of final reach direction used to separate learners and non-learners in **Experiment 1**.
(PDF)

S2 Data Trial-by-trial analyses. Trial-by-trial analyses that examine: a) behavioural estimates of motor and exploratory contributions to movement variability, and b) movement variability as a function of reinforcement history.
(PDF)

S3 Data Best-fit parameters of the learning model. Finds the best-fit parameters of the learning model using bootstrapping to minimize squared error.
(PDF)

S4 Data Influence of initial reward probability on learning rate. Simulations to demonstrate that the initial reward probability of the shallow landscape has a marginal influence on learning rate.
(PDF)

S1 Fig Distribution of final reach direction. The frequency (*y*-axis) of final reach position (*x*-axis) for the 80 participants collected in **Experiment 1**. We used a z-score cutoff of 1.0 (dashed, vertical black line) to separate the learners (z-score ≥ 1.0) from the non-learners (z-score < 1.0).
(PDF)

S2 Fig Movement variability as a function of reinforcement history. Average standard deviation of changes in reach angle between trials *n* and *n* + 1 (*y*-axis) given reinforcement history (*x*-axis) for **A)** the behaviour data of participants in **Experiment 1**, and **B)** the learning model simulations. Base and Wash represent baseline (trials 25-50) and washout (trials 400-450), respectively. The best-fit parameters (*x*-axis) and their magnitudes (*y*-axis) of the variability state-space model developed by Pekny and Colleagues (2015) as applied to **C)** the behavioural data of participants in **Experiment 1** and **D)** the outputs of our learning model. For all subplots, the orange and blue colours represent participants (or model simulations) that experienced the shallow or steep reinforcement landscapes, respectively. Grey represents the average collapsed across all participants or simulations. Error bars are ±1.0 SE.
(PDF)
