# Supplementary material for: The gradient of the reinforcement landscape influences sensorimotor learning
Source: PLoS Comput Biol. 2019 Mar 4;15(3):e1006839. doi: 10.1371/journal.pcbi.1006839 (PMC6417747; doi:10.1371/journal.pcbi.1006839)
Supplement: S1 Data — Cutoff criterion of final reach direction used to separate learners and non-learners in Experiment 1. (PDF) [file pcbi.1006839.s001.pdf]

S1 DATA

Cutoff Criterion Used to Separate Learners and Non-Learners

For each experimental group in *Experiment 1*, there were two distinct subpopulations of participants: those who changed their reaching direction during the experimental trials and ascended the reinforcement landscape (learners) and those who did not change their reaching behaviour during the experimental trials (non-learners). For each participant, we determined their average reach angle during the last 100 trials of the experimental trials. Plotting a histogram of participant final positions revealed a bimodal distribution, representing the two subpopulations of learners and non-learners (see **Fig. S1**).

We found that a $z\text{-}score$ of 1.0 was a suitable cutoff for separating the learners from the non-learners. That is, participants whose final reach position had a $z\text{-}score \geq 1.0$ were classified as learners, and those whose final reach position had a $z\text{-}score < 1.0$ were classified as non-learners. Although the non-learners affect the average final reach angle of their respective groups, they did not bias the time-constant (λ) of the exponential curves. In other words, the non-learners do not affect the time taken to reach asymptotic behaviour. Crucially, those experiencing a steep or shallow reinforcement landscape had significantly different learning rates, irrespective of whether non-learners were ($p = 0.021$) or were not ($p = 0.012$) included in the comparison.

Moreover, the same cutoff was used in *Experiment 2* to separate steep learners, shallow learners and non-learners. For both the steep clockwise and steep counter-clockwise reinforcement landscapes, participants that moved at least 1.0 $z\text{-}score$ away from baseline in the direction of the steep slope were classified as steep learners. Participants that moved at least 1.0 $z\text{-}score$ away from baseline in the direction of the shallow slope were classified as shallow learners. Those who stayed within $\pm\, 1.0$ $z\text{-}score$ relative to baseline behaviour were classified as non-learners.
