# Supplementary material for: The gradient of the reinforcement landscape influences sensorimotor learning
Source: PLoS Comput Biol. 2019 Mar 4;15(3):e1006839. doi: 10.1371/journal.pcbi.1006839 (PMC6417747; doi:10.1371/journal.pcbi.1006839)
Supplement: S2 Data — Trial-by-trial analyses that examine: a) behavioural estimates of motor and exploratory contributions to movement variability, and b) movement variability as a function of reinforcement history. (PDF) [file pcbi.1006839.s002.pdf]

S2 DATA

Trial-by-Trial Analyses

A) Behavioural estimates of motor and exploratory contributions to movement variability

There is evidence that movement variability is greater following an unsuccessful action compared to following a successful action (Izawa and Shadmehr, 2011; Galea et al., 2013; Pekny et al., 2015). Accordingly, an important aspect of our model was estimating movement variability following a successful reach and an unsuccessful reach.

As a reminder, our model assumed that the movement variability (σ^2_{n+1}) after a successful reach ($r = 1$) was the result of motor execution noise (σ_m). We also assumed that movement variability (σ^2_{n+1}) after an unsuccessful action ($r = 0$) is the result of the additive effects of motor noise (σ_m) and exploratory noise (σ_e). Thus, as shown in **Eq. 1** in the manuscript, movement variability can be modelled as

$$\sigma^2_{n+1} = \begin{cases} \sigma^2_m & r = 1 \qquad (B1a), \\ \sigma^2_m + \sigma^2_e & r = 0 \qquad (B1b). \end{cases}$$

Using the behavioural data from participants in *Experiment 1* we estimated (σ_m) and (σ_e) using a trial-by-trial analysis.

Following the work of Pekny and colleagues (2015), we calculated trial-by-trial angular differences ($\Delta\theta$) by

$$\Delta\theta = \theta_{n+1} - \theta_n \qquad (B2).$$

By iterating **Eq. B2** over many trials we were able to build a distribution of $\Delta\theta$. From this distribution one can estimate the variance of $\Delta\theta$, that is $\sigma^2_{\Delta\theta}$.

It is important to consider, however, that both θ_{n+1} and θ_n are independent random processes and their respective variances, $\sigma^2_{\theta_{n+1}}$ and $\sigma^2_{\theta_n}$, are additive in determining $\sigma^2_{\Delta\theta}$.

This is summarized by

$$\sigma^2_{\Delta\theta} = \sigma^2_{\theta_{n+1}} + \sigma^2_{\theta_n} \quad (B3).$$

As discussed later, the variables in this equation are estimated from a distribution by repeatedly performing **Eq. B2** across many trials. For our model, we wanted to isolate the movement variability on a single-trial (not the additive variance that results from subtracting two successive trials). Assuming that $\sigma^2_{\theta_{n+1}}$ and $\sigma^2_{\theta_n}$ are approximately similar in magnitude and then following some simple rearranging of **Eq. B3**, we approximated single-trial movement variance (σ^2_θ) with

$$\sigma^2_\theta \approx \frac{\sigma^2_{\Delta\theta}}{2} \quad (B4).$$

As described in further detail below, we then used **Eqs. B1-B4** to estimate motor noise (σ_m) and exploratory noise (σ_e).

We used **Eqs. B1a, B2-B4** on the last 25 baseline trials to estimate motor noise (σ_m). Using baseline trials to estimate motor noise, rather than calculating changes in reach angle after a successful action, allowed us to eliminate the confound of updated reach aim on trial $n+1$ following a rewarded on trial n. That is, we wanted to be assured that the angular differences were caused by motor noise alone, and not confounded with changes in reach angle due to an updating of reach aim following a successful action. We related motor noise and single-trial baseline variance using **Eqs. C1a** and **C4**, such that

$$\sigma^2_\theta \approx \sigma^2_m \quad (B5).$$

Using *Experiment 1* participants, our experimental estimate of σ_m was $0.85(0.83, 0.87)$ $z\text{-}score$.

To estimate the additive of effects of motor noise (σ_m) and exploratory noise (σ_e) following an unsuccessful trial (see **Eq. 1b**), we used **Eqs. B2-B4** when trials n and $n-1$

were unrewarded. This was done to minimize the differences in magnitude between $\sigma^2_{\theta_{n+1}}$ and $\sigma^2_{\theta_n}$. Using **Eqs. B1b** and **B4**, we can then relate single-trial variability following an unsuccessful trial to the additive effects of both motor and exploratory movement variability with

$$\sigma^2_\theta \approx \sigma^2_m + \sigma^2_e \quad (B6).$$

Using **Eqs. B2-B4** we found that the estimate of σ^2_θ following unsuccessful trials was $1.31(1.25, 1.37)$ z-$score$. Given we had an estimate of both σ^2_θ and σ_m, we rearranged **Eq. B6** to isolate σ_e. As a result, we estimated σ_e to have a magnitude of approximately $0.99(0.93, 1.05)$ z-$score$.

The experimental estimates of $\sigma_m(0.85)$ and $\sigma_e(0.99)$ were used as initial guesses during the bootstrap optimization best-fit procedure (see **S3 DATA**). Since these estimates are based on the assumptions we list above, we let both σ_m and σ_e free to vary in Step 2 of the bootstrap optimization best-fit procedure. We found that the best-fit values (and corresponding 95th percentile confidence intervals) of α, σ_m, and σ_e were $0.40(0.25, 0.63), 0.9(0.813, 1.02)$, and $0.81(0.63, 0.97)$, respectively. Moreover, there was an overlap of the 95th percentile confidence intervals between the experimental estimates of variance and the corresponding best-fit parameters found from the bootstrap optimization best-fitting procedure.

B) Trial-by-trial analysis based on reinforcement history

Using methods described by Pekny and colleagues (2015), we examine movement variability as a function of previous reward history. The first analysis examines the variance of trial-by-trial changes in reach angle given the reward of the last previous trials. The second analysis finds the coefficients of a variability state-space model. This model attempts to predict movement variability given the successful ($r = 1$) and unsuccessful ($r = 0$) reaches of the previous three trials.

For the first analysis, we used **Eq. B2** to calculate the trial-by-trial changes in reach angle for each combination of successful and unsuccessful reaches during the previous three trials. This resulted in 8 different combinations since we examined the previous three trials. In addition to experimental trials, we also calculated trial-by-trial changes in reach angle during baseline and washout. Similar to Pekny and colleagues (2015), we then calculated the standard deviation of these trial-by-trial changes in reach angle.

For the second analysis, we used the following variability state-space model developed by Pekny and colleagues (2015):

$$|\Delta\theta| = \alpha_0(1 - r(n)) + \alpha_1(1 - r(n-1)) + \alpha_2(1 - r(n-2)) + \epsilon \quad (B7).$$

Here, the absolute change in reach angle ($\Delta\theta$) between trials n and $n+1$ is a function of previous unsuccessful reaches. In the equation above, the $r(\cdot)$ terms are set to 1 if the nth reach is successful and set to 0 if the nth reach is unsuccessful. Each α_j coefficients dictate the amount of additional movement variability on trial $n+1$ given a corresponding unsuccessful reach during one of the previous three trials. ϵ represents the unexplained variance due to unsuccessful reaches. It has been interpreted as the amount of movement variability due to motor noise (Pekny et al., 2015).

We performed both of these analyses on the behavioural data of *Experiment 1*. We also performed these analyses on the output of our learning model. Using our model, we simulated $10,000$ 'individuals' experiencing the shallow reinforcement landscape and another $10,000$ 'individuals' experiencing the steep reinforcement landscape.

Since we did not did not expect differences between conditions, we initially analyzed participants' data irrespective of the experienced reinforcement landscape. For qualitative comparison, we then performed the same analysis after partitioning participants according to whether they experienced either the steep or shallow reinforcement landscape. The same procedure was performed when analyzing the outputs of our learn-

ing model. To compare to our results, we encourage the reader to examine Fig. 5 in the paper by Pekny and colleagues (2015).

The variance of trial-by-trial changes in reach angle as a function of previous reinforcement history is displayed in **S2 Figure**. Our behavioural results (**S2,A Figure**) are quite similar to those reported by Pekny and collleagues (2015; see Fig. 5A). Specifically, we also found that the variance of trial-by-trial changes in reach angle was lower following a successful trial than an unsuccessful trial. Unexpectedly, we also observed a qualitative difference between participants experiencing the steep and shallow reinforcement landscapes. However, as mentioned by Dhawale and colleagues (2017) and expanded upon below, using the variance of trial-by-trial changes in reach angle may not be well suited in truly capturing a reward-prediction error.

The results of performing the trial-by-trial difference analysis on the outputs of our learning model are displayed in **S2,B Figure**. In terms of shape, the patterns are surprisingly similar to the empirical results of both our work (**S2,A Figure**) and Pekny and colleagues (2015). This was unexpected given that our learning model considers only the reinforcement state of the previous trial when modulating movement variability. That is, given the formulation of our model we did not expect reinforcement two or three trials back to have an impact on movement variability on a current trial. However, as suggested by Dhawale et al., 2017, there are potential confounds when trying to relate the variance of trial-by-trial changes in reach angle to previous reinforcement history. Some potential factors beyond reinforcement history that may influence the variance of trial-by-trial changes in reach angle are: a) updates in reach aim, b) independent noise between trials, or c) the propagation of dependent noise across successive trial (van Beers et al., 2013, Chaisanguanthum et al., 2014). As highlighted by the analysis of trial-by-trial changes in reach angle on our model outputs, it is difficult to solely attribute changes in movement variability with previous reinforcement history.

The learning model did well to mimic the average variance of trial-by-trial changes in reach angle given reinforcement history. However, it did not seem to capture apparent differences between participants experiencing a steep reinforcement landscape and those experiencing a shallow reinforcement landscape. It is possible that these apparent differences are due to a reward prediction error. However, this is difficult to determine given the aforementioned limitations when examining the variance of trial-by-trial changes in reach angle given recent reinforcement history.

We also examined the parameters (α_j and ϵ) of a variability state-space model (Pekny et al., 2015) that were fit to our *Experiment 1* data (**S2,C Figure**) and to the outputs of our learning model when simulating *Experiment 1* (**S2,D Figure**). We found that the best-fit parameters of the variability state-space model were strikingly similar between our behavioural data and the outputs of our learning model. Moreover, they were also quite similar to the findings of Pekny and colleagues (2015; see Fig. 5B). The interpretation of these results mirrors that of the trial-by-trial analysis above. Moreover, the same potential confounds that may influence the ability to relate the variance of trial-by-trial changes in reach angle to reinforcement history would also apply to this variability state-space model.

Despite the potential limitations of relating trial-by-trial changes in reach angle to reinforcement history, the reported results above highlight that our model did well to capture the average variance of trial-by-trial changes in reach angle. Moreover, these results demonstrated that we made reasonable estimates of motor (σ_m) and exploratory (σ_e) contributions to movement variability.

REFERENCES

1. Chaisanguanthum, K. S., Shen, H. H., & Sabes, P. N. (2014). Motor variability arises from a slow random walk in neural state. Journal of Neuroscience, 34(36), 12071-12080.
