# Supplementary material for: The gradient of the reinforcement landscape influences sensorimotor learning
Source: PLoS Comput Biol. 2019 Mar 4;15(3):e1006839. doi: 10.1371/journal.pcbi.1006839 (PMC6417747; doi:10.1371/journal.pcbi.1006839)
Supplement: S3 Data — Finds the best-fit parameters of the learning model using bootstrapping to minimize squared error. (PDF) [file pcbi.1006839.s003.pdf]

S3 DATA

Best-fit Parameters of the Learning model

The reinforcement-based state-space model has three free parameters: α, σ_m, and σ_e. To find the best-fit parameters and their corresponding confidence intervals, we performed a bootstrap optimization fitting procedure. Fitting was done using a two-step process. For all optimizations, we used a Nelder-Mead optimization routine contained in the SciPy module (optimize, minimization) in Python.

<u>Step 1: Finding Initial Guesses with Behavioural Estimates and 'Warm-start' Optimization</u>

We used a 'warm-start' bootstrap optimization procedure to find an initial guess of α, where σ_m and σ_e were held as constants (0.85 and 0.99, respectively). Estimates of σ_m and σ_e were found using a trial-by-trial analysis (see **S2 DATA**).

For each bootstrap ($N = 1000$), we resampled with replacement separately for participants experiencing a steep landscape and participants experiencing a shallow landscape. For each group, we averaged across participants to find the mean reach angle of each trial. Then we fit an exponential function (**Eq. 6**) to these two experimental groups. Consequently, we obtained a time-constant that defines the rate of learning for participants experiencing the steep reinforcement landscape ($\lambda_{steep}^{behaviour}$) and a time-constant for participants experiencing the shallow reinforcement landscape ($\lambda_{shallow}^{behaviour}$). For each exponential function, we also found the best-fit term that defines the asymptotic reach angle (a).

Simultaneously, with our learning model (**Eq. 1**) we simulated 2000 'individuals' experiencing the steep reinforcement landscape and another 2000 'individuals' experiencing the shallow reinforcement landscape. Like the behavioural data, we averaged across simulated individuals to obtain the mean reach angle for each trial. We then fit an exponential function to the simulated individuals that experienced the steep reinforcement landscape and the simulated individuals that experienced a shallow reinforcement

landscape. This yielded a time-constant that defines the rate of learning for simulated individuals experiencing the steep reinforcement landscape (λ_{steep}^{model}) and a time-constant for simulated individuals experiencing the shallow reinforcement landscape ($\lambda_{shallow}^{model}$). We also found the best-fit term that defines the asymptotic reach angle (a) for each exponential function.

For each bootstrap (i), we then found the best-fit α^i by minimizing error between the behavioural time-constants and model time-constants according to following equation:

$$\alpha^i = \underset{\alpha}{\arg\min}\{|(\lambda_{steep}^{behaviour} - \lambda_{steep}^{model})|^2 + |(\lambda_{shallow}^{behaviour} - \lambda_{shallow}^{model})|^2\} \qquad (C1).$$

This process produces a single best-fit α^i per bootstrap.

Repeating this process 1000 times yields a distribution of α_i. We then took the mean of this distribution to find a single α, which was then used as the initial guess for the best-fit optimization procedure described below.

Step 2: Finding the Best-fit parameters and their 95th percentile confidence intervals

The procedure for finding the best-fit parameters was the same as described above, except that all three parameters (α, σ_m, σ_e) of the model were free to vary:

$$\alpha^{opt_i}, \sigma_m^{opt_i}, \sigma_e^{opt_i} = \underset{\alpha, \sigma_m, \sigma_e}{\arg\min}\{|(\lambda_{steep}^{behaviour} - \lambda_{steep}^{model})|^2 + |(\lambda_{shallow}^{behaviour} - \lambda_{shallow}^{model})|^2\} \qquad (C2).$$

From each of the resulting parameter distributions, we used the mean as the best-fit value for each parameter. Further, an advantage of this bootstrap optimization procedure is that confidence intervals can be found from these parameter distributions. The best-fit values (and corresponding 95th percentile confidence intervals) of α, σ_m, and σ_e were $0.40(0.25, 0.63)$, $0.9(0.813, 1.02)$, and $0.81(0.63, 0.97)$, respectively.
