# Supplementary material for: The gradient of the reinforcement landscape influences sensorimotor learning
Source: PLoS Comput Biol. 2019 Mar 4;15(3):e1006839. doi: 10.1371/journal.pcbi.1006839 (PMC6417747; doi:10.1371/journal.pcbi.1006839)
Supplement: S4 Data — Simulations to demonstrate that the initial reward probability of the shallow landscape has a marginal influence on learning rate. (PDF) [file pcbi.1006839.s004.pdf]

S4 DATA

Influence of Initial Reward Probability on Learning Rate

Here we performed a simulation with our learning model ($n = 100,000$) to explore the influence of initial reward probability on the rate of learning. Specifically, we examined the learning rate when experiencing a shallow landscape with an initial reward probability (z-$score = 0$) set to 33.3% (**Fig. 1B)** or 66.7%. Shifting the initial reward probability up to 66.7% resulted in a maximal reward probability of 100.0%. We found that changing the initial reward probability from 33.3% to 66.7% resulted in a marginally slower learning rate (i.e., λ changed from 49.6 to 55.9). These marginal learning rate changes can be attributed to a slightly lower frequency of additional exploratory variability. Nevertheless, our simulations find that a steep landscape results in faster learning ($\lambda = 28.0$; **see Results**) compared to a shallow landscape, irrespective of the latter's initial reward probability.
