# Supplementary figures and images for: The gradient of the reinforcement landscape influences sensorimotor learning
Source: PLoS Comput Biol. 2019 Mar 4;15(3):e1006839. doi: 10.1371/journal.pcbi.1006839 (PMC6417747; doi:10.1371/journal.pcbi.1006839)

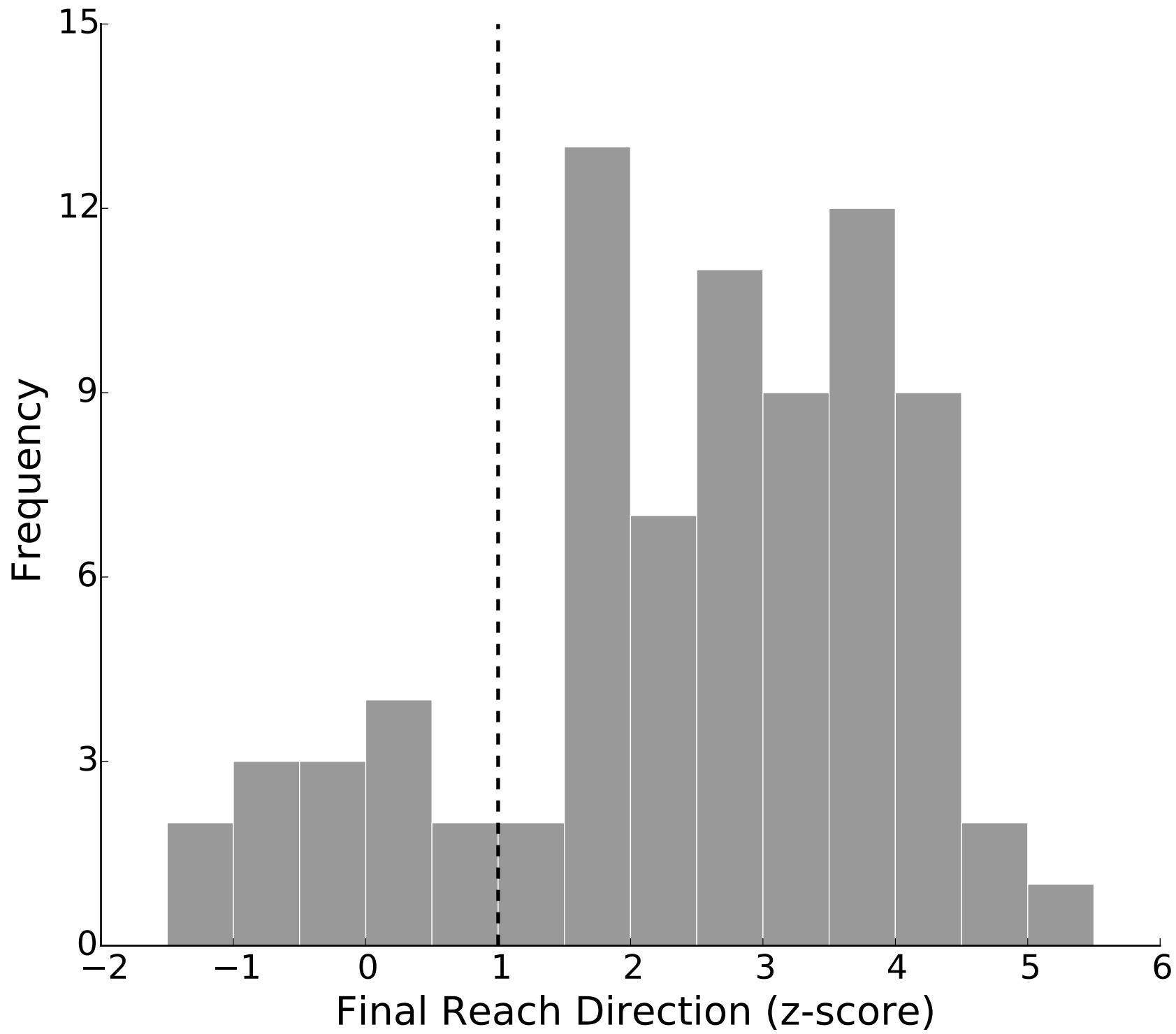

Supplement: S1 Fig — The frequency (y-axis) of final reach position (x-axis) for the 80 participants collected in Experiment 1. We used a z-score cutoff of 1.0 (dashed, vertical black line) to separate the learners (z-score ≥ 1.0) from the non-learners (z-score < 1.0). (PDF) [file pcbi.1006839.s005.pdf]

**A**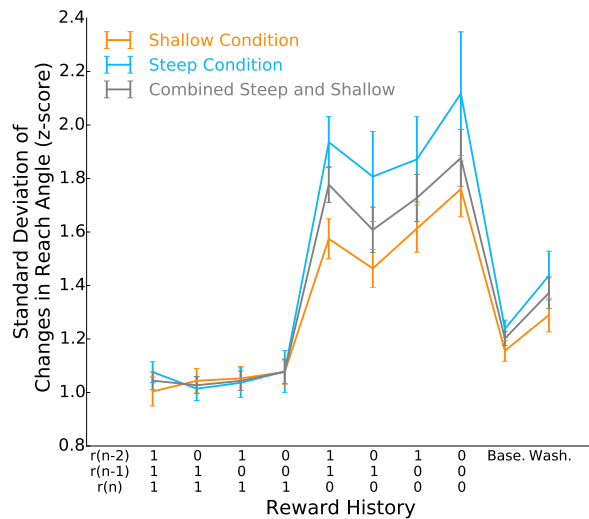**B**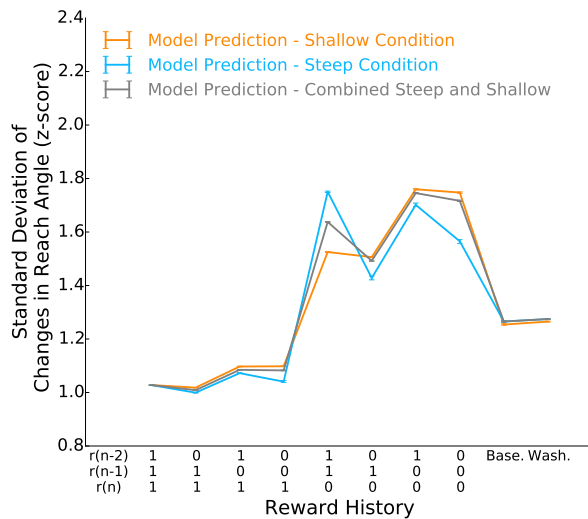**C**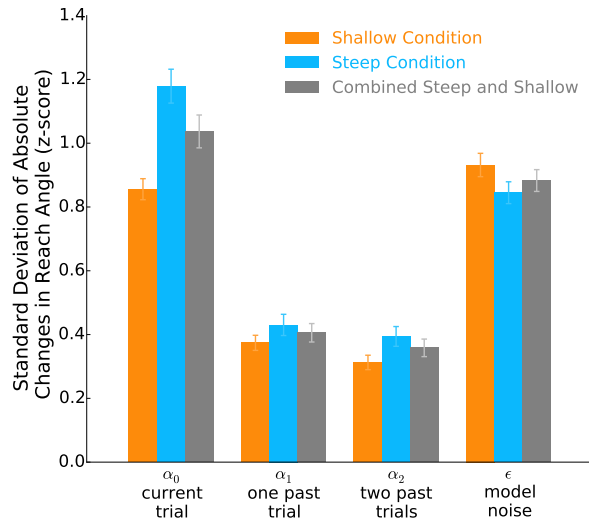**D**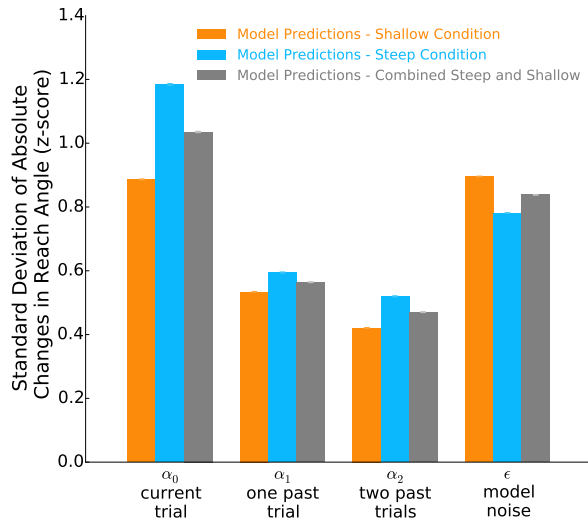

Supplement: S2 Fig — Average standard deviation of changes in reach angle between trials n and n + 1 (y-axis) given reinforcement history (x-axis) for A) the behaviour data of participants in Experiment 1, and B) the learning model simulations. Base and Wash represent baseline (trials 25-50) and washout (trials 400-450), respectively. The best-fit parameters (x-axis) and their magnitudes (y-axis) of the variability state-space model developed by Pekny and Colleagues (2015) as applied to C) the behavioural data of participants in Experiment 1 and D) the outputs of our learning model. For all subplots, the orange and blue colours represent participants (or model simulations) that experienced the shallow or steep reinforcement landscapes, respectively. Grey represents the average collapsed across all participants or simulations. Error bars are ±1.0 SE. (PDF) [file pcbi.1006839.s006.pdf]
